# Clove Oil (*Syzygium aromaticum* L.) Activity against *Alicyclobacillus acidoterrestris* Biofilm on Technical Surfaces

**DOI:** 10.3390/molecules25153334

**Published:** 2020-07-22

**Authors:** Alina Kunicka-Styczyńska, Agnieszka Tyfa, Dariusz Laskowski, Aleksandra Plucińska, Katarzyna Rajkowska, Krystyna Kowal

**Affiliations:** 1 Institute of Fermentation Technology and Microbiology, Faculty of Biotechnology and Food Sciences, Lodz University of Technology, Wólczańska Str. 171/173, 90-924 Łódź, Poland; tyfa.agniesia@gmail.com (A.T.); aleksandra.plucinska@dokt.p.lodz.pl (A.P.); katarzyna.rajkowska@p.lodz.pl (K.R.); krystyna.kowal@p.lodz.pl (K.K.); 2 Department of Microbiology, Faculty of Biological and Veterinary Sciences, Nicolaus Copernicus University in Toruń, Lwowska Str. 1, 87-100 Toruń, Poland; laskosd@umk.pl

**Keywords:** clove oil, antibiofilm, *Alicyclobacillus acidoterrestris*, bacterial biofilm, glass, PVC

## Abstract

Acidotermophilic bacteria *Alicyclobacillus acidoterrestris* is one of the main contaminants in the fruit industry forming biofilms which are difficult to remove from the production line by conventional methods. An alternative approach aims for the use of essential oils to prevent *Alicyclobacillus* biofilm development. The effect of clove essential oil on *A. acidoterrestris* biofilms on glass and polyvinyl chloride surfaces under static and agitated culture conditions was investigated by atomic force microscopy and the plate count method. The medium-flow and the type of technical surface significantly influenced *A. acidoterrestris* biofilm. The PVC was colonized in a greater extent comparing to glass. Clove essential oil in 0.05% (*v/v*) caused 25.1–65.0% reduction of biofilms on the technical surfaces along with substantial changes in their morphology by a decrease in the biofilm: height, surface roughness, and surface area difference. The oil also induced alteration in individual bacterial cells length and visible increase of their roughness. Clove essential oil seems to release EPS from biofilm and thus induce detachment of bacteria from the surface. Due to anti-*A. acidoterrestris* biofilm activity, the clove oil may be used in the juice industry to hinder a development of *A. acidoterrestris* biofilms on production surfaces.

## 1. Introduction

Bacteria are reported to grow on both biotic and abiotic surfaces in the form of matrix-enclosed homogenous and/or heterogeneous populations recognized as biofilms. Biofilm formation is considered as a multistep process in which cells adhesion and colonization of a particular surface occur [1,2]. Subsequent production of the extracellular polymeric substances (EPS) stabilizes the biofilm and provides an elevated level of bacterial protection against unfavorable conditions together with a higher resistance to chemical substances [3,4]. Specific structure and nature of biofilms stimulate a constant up- and down-regulation of certain genes, followed by metabolism reduction and diversification of bacterial cells functioning. Moreover, high density of biofilm enhances cells detachment, their mitigation, and hence the colonization of new locations. Despite a wide knowledge and a variety of elimination techniques, biofilms still remain a great concern for the food industry [5,6,7,8]. Biofilm composition varies depending on the particular branch of industry; however, little is known about biofilms formed by *Alicyclobacillus acidoterrestris*.

This bacterium is an aerobic and Gram-positive microorganism, commonly found in soil, on the surface of fruits and at several steps of fruit juices processing [9,10,11,12]. *A. acidoterrestris* is able to withstand harsh conditions, including a wide range of pH (2.0–6.0) and temperature (20–60 °C). Additionally, spore production enables survival of pasteurization and their outgrowth during the storage of fruit juices, hence why *A. acidoterrestris* is regarded as a main threat for fruit juice industry which may contribute to considerable economic losses. Apart from planktonic form, *A. acidoterrestris* is known to colonize variety of technical materials (stainless steel, nylon, glass, PVC, polystyrene) and hence to form biofilms [13,14,15]; however, knowledge on the elimination of their biofilms is limited.

Clove oil expresses a strong antimicrobial activity. Its minimum inhibitory concentrations against bacteria *Staphylococcus aureus*, *Staphylococcus epidermidis*, *Escherichia coli*, and *Pseudomonas aeruginosa* were noted in the range of 0.2–0.625 mg/mL [16,17,18]. The antibacterial effect is assigned to its main compound eugenol interactions with bacterial cell membranes and disruption of DNA synthesis [18]. The antifungal activity of the clove oil was proved against a variety of yeast and moulds: *Candida albicans*, *Candida parapsilosis*, *Candida krusei*, *Alternaria* sp., *Aspergillus niger*, *Aspergillus fumigatus*, *Penicillum* sp., *Microsporum canis*, *Microsporum gypseum*, *Trichophyton rubrum*, *Trichophyton mentagrophytes,* and *Epidermophyton floccosum* [19,20]. The antifungal activity of clove oil is attributed to a decrease of ergosterol synthesis, the component of the fungal cell wall [20]. Clove oil at the concentrations in the range of 2.5–10.0% was also effective against food contaminants *Bacillus cereus*, *Bacillus subtilis*, *Staphylococcus* sp., *E. coli*, *Aspergillus* sp., *Penicillium* sp., and *Rhizopus* sp. [19]. The oil action against food borne pathogens *Listeria monocytogenes*, *S. aureus*, *E. coli* O157:H7, and *Salmonella* sp. was also confirmed [21]. The vivid antibiofilm effect of clove oil was observed against *E. coli* O157:H7 [22], *P. aeruginosa,* and *Aeromonas hydrophila* [23]. The clove oil even in its sub-MICs concentrations substantially inhibited the bacterial biofilm formation and reduced virulence factors of the pathogens. Interestingly, solid liposomes of clove oil applied on raw vegetables exhibited anti-biofilm activity against *E. coli* O157:H7 without any adverse effects on sensory quality of food [24]. According to the US Food and Drug Administration clove essential oil was approved generally recognized as safe (GRAS) to use as a food additive and in dentistry [25].

The possible use of essential oil as a disinfectant promotes substances containing the active ingredient at a high concentration, regardless of the origin of the essential oil. The eugenol content noted from 63.8 to 84.8% [26] and its strong antibacterial activity [27] make the clove oil a good candidate for the anti-biofilm agent. Furthermore, the clove essential oil organoleptic profile is congruent with many fruit juice taste and aromas. According to current studies, a wide range of chemical compounds and substances of natural origin (essential oil’s components, bacteriocins) are reported to inhibit *A. acidoterrestris* growth and reduce the number of spores both in growth media and fruit juices [28,29,30,31,32,33,34]. Considering *A. acidoterrestris* biofilm formation, the only sanitizers displaying effectiveness in the removal of biofilm from industrial environments are peracetic acid, sodium hypochlorite, and quaternary ammonia salts [13]. There are scarce reports outlining the impact of natural substances on *A. acidoterrestris* biofilm formation and its removal from technical surfaces. Moreover, the morphological changes of *Alicyclobacillus* biofilms formed on the technical materials are not extensively researched. The main objective of this study was to investigate the antibiofilm activity of clove essential oil against *A. acidoterrestris* on glass and polyvinyl chloride surfaces. The oil effect on the level and morphology of biofilms both in static cultures and the cultures subjected to agitation were studied.

## 2. Results

The *Syzygium aromaticum* (L.) Merr. and Perry buds oil composition was (%): eugenol 86.99; α-copaene 0.07; β-caryophyllene 8.76; cadina-1(6),4-diene 0.04; α-humulene 1.91; ɣ-muurolene 0.02; β-selinene 0.02; α-selinene 0.05; β-farnesene 0.06; (Z)-calamenene 0.11; δ-cadinene 0.27; cadina-1,4-diene 0.03; and humulene epoxide 0.11 (Table 1). The quantity of β-caryophyllene and eugenol met the requirements of clove oils acceptable by European Pharmacopeia, even though the presence of acetyleugenol was not detected [35]. According to our previous research (data not published), the minimum inhibitory concentration of clove essential oil for both tested *A. acidoterrestris* strains in the planktonic form was 0.05% (*v/v*) and, therefore, this concentration was used in the antimicrobial testing on mature *A. acidoterrestris* biofilms.

**Figure 1:**
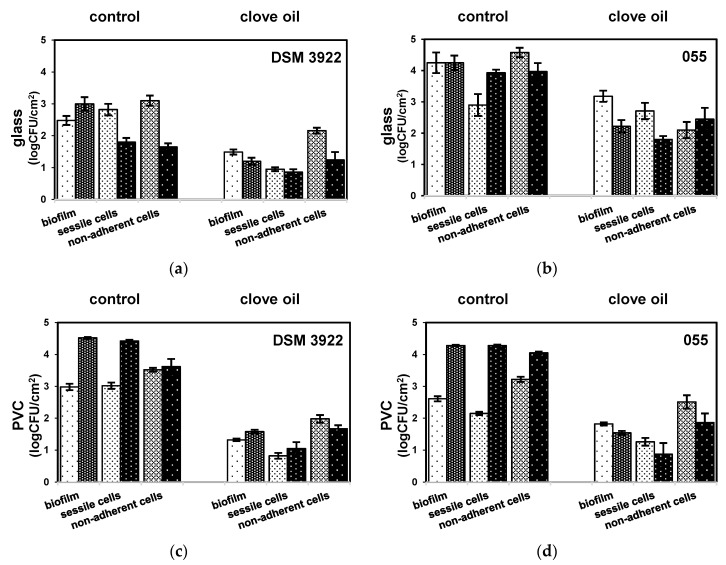
Quantification of *Alicyclobacillus acidoterrestris* biofilm formed on glass and polyvinyl chloride (PVC) surfaces in cultures non-agitated (light bars) and agitated (dark bars) without clove oil (control) and with 0.05% clove oil: (**a**) *A. acidoterrestris* DSM 3922 on glass surface; (**b**) *A. acidoterrestris* 055 on glass surface; (**c**) *A. acidoterrestris* DSM 3922 on PVC surface; and (**d**) *A. acidoterrestris* 055 on PVC surface.

*A. acidoterrestris* DSM 3922 reference strain and environmental strain *A. acidoterrestris* 055 formed biofilm on the surface of glass and polyvinyl chloride in agitated and non-agitated cultures. According to our previous study, the 72-h incubation period was sufficient enough for *A. acidoterrestris* to generate a mature biofilm [15]. Both strains exhibit an ability to form a biofilm with different efficiency, dependent on the culture conditions and the surface material. The level of biofilm in the cultures without the clove oil ranged from 2.48 log CFU/cm^2^ to 4.52 log CFU/cm^2^ (Figure 1). Simultaneously, agitation promoted the *A. acidoterrestris DSM 3922* biofilm development on both technical surfaces and *A. acidoterrestris* 055 on PVC increasing the level in 20.9–39.0%. The addition of clove oil resulted in a substantial reduction of biofilm formation (1.20–3.18 log CFU/cm^2^) and its level was decreased in 25.1–55.7% and 47.7–65.0% in non-agitated and agitated cultures, respectively. Interestingly, the composition of the biofilm measured as the ratio of non-adherent cells to sessile cells changed at the presence of clove oil and a nearly double increase in the number of non-adherent cells compared to sessile cells was noted in regard to the culture without the oil (Figure 1). The ratio of cells loosely attached classified as non-adherent to adherent recognized as sessile ranged from 0.87 to 1.57 in the biofilms formed by both strains on glass and PVC in all growth cultures without the clove oil. The ratio for biofilms created with the suppression of the oil was in the range of 1.99–2.41 with one exception of *A. acidoterrestris* 055 biofilm in the non-agitated cultures formed on glass (0.78).

**Figure 2:**
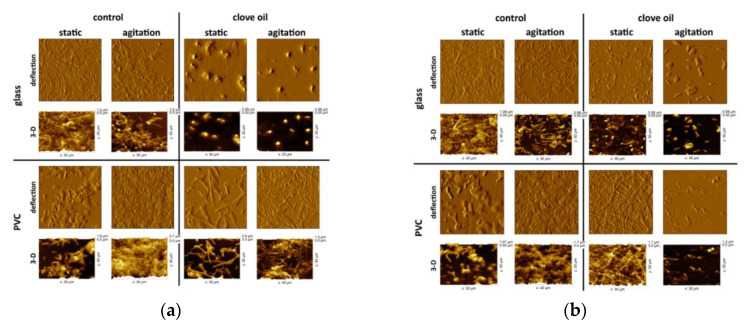
AFM imaging of *A. acidoterrestris* biofilm formed on glass and polyvinyl chloride (PVC) surfaces in cultures non-agitated (static) and agitated without clove oil (control) and with 0.05% clove oil: (**a**) *A. acidoterrestris* DSM 3922; (**b**) *A. acidoterrestris* 055.

**Figure 3:**
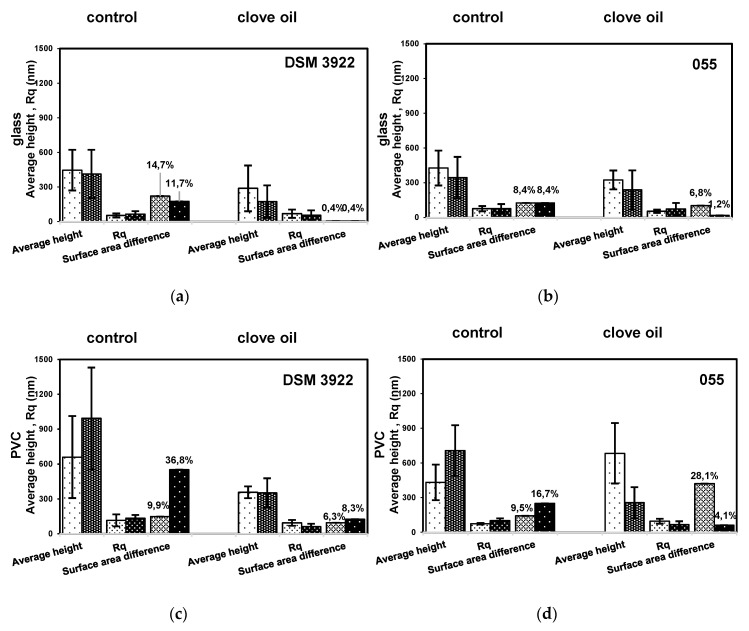
Structural parameters of *A. acidoterrestris* biofilm formed on glass and polyvinyl chloride (PVC) surfaces in cultures non-agitated (light bars) and agitated (dark bars) without clove oil (control) and with 0.05% clove oil: (**a**) *A. acidoterrestris* DSM 3922 on glass surface; (**b**) *A. acidoterrestris* 055 on glass surface; (**c**) *A. acidoterrestris* DSM 3922 on PVC surface; (**d**) *A. acidoterrestris* 055 on PVC surface.

Figure 2 shows the morphology of the biofilm formed on glass and polyvinyl chloride in the presence or absence of 0.05% clove oil. On the glass surface and under agitation, *A. acidoterrestris* DSM 3922 vegetative cells and spores are aggregated with extracellular polymer substances (EPS), while biofilm formed by *A. acidoterrestris* 055 was rather composed of vegetative cells surrounded by an EPS layer. In general, in static conditions (non-agitated), biofilm formed a dense three-dimensional structure on the glass surface, while biofilm grown under agitation formed a two-dimensional layer with areas devoid of cells. In contrast, biofilm formed on polyvinyl chloride surface was denser under agitation then under static conditions. Regardless of the applied conditions, the polyvinyl chloride surface was preferably colonized by the strains. However, the strains formed a highest biofilm with the most irregular structure (surface area difference) on polyvinyl chloride surface under shaking conditions. The average heights and biofilm surface area difference for reference strain were 992.6 nm and 36.8% while for the environmental strain 708.2 nm and 16.7%, respectively (Figure 3). The results indicated that the agitation has more effect on development of biofilm on the surface of polyvinyl chloride than glass irrespective of the clove oil presence. As it was noted the agitation substantially increases the biofilm level on PVC in the cultures without the clove oil and is accompanied by the high surface area difference. The differential structure of the surface of the biofilm may be associated with micro-caves formation enhancing the cells contact with the growth medium.

The AFM imaging reveals differences in biofilm structures formed by *A. acidoterrestris* strains induced by clove essential oil. In general, strains colonize the material’s surfaces in the form of aggregates or poorly developed biofilm with a small amount of EPS. However, in contrast to cultures without the oil, these aggregates contained greater amount of spores (Figure 2). The treated biofilms have lower average height, surface roughness and surface area difference in contrast to pristine biofilm. The only exception was 055 strain on PVC in non-agitated culture forming 33% higher biofilm but with three times higher surface area difference. These signify formation of large, flat areas without cells and EPS. The height of biofilm decreased by 35% to 64% depending on the experiment variant and strains (Figure 3). However, it seems that agitation conditions with clove oil stimulated to a greater extent a detachment of bacteria from surfaces.

On the contrary, biofilm formed by environmental strain on PVC surface in a static culture was higher by 36% in the presence of clove oil. However, this could be explained by nonhomogeneous biofilm structure and various extent of surface colonization by the bacteria. Moreover, clove essential oil may not only release the EPS from biofilm inducing detachment of bacteria from surface, but also affect the adherence capability of cells.

Furthermore, clove oil displayed an activity towards *A. acidoterrestris* cells resulting in changes in morphology. Clove treatment led to either change in the cells length or the deformation of cell wall surface with visible increase of its roughness.

## 3. Discussion

Studies concerning acidothermophilic bacteria are of great interest and applicable value for juice industry as spores formation and subsequent their germination increases probability of bacterial contamination at the final production steps and products. The control and elimination of *Alicyclobacillus* biofilms should be adapted to the needs of the production processes [36].

However, there are some reports concerning *Alicyclobacillus’* ability to form biofilms [13,14,36,37], as the *A. acidoterrestris* biofilm morphology is still poorly recognized. The literature lacks information on anti-biofilm action of essential oils on these bacteria. To our knowledge, some research describes the activity of lemon essential oil towards *A. acidoterrestris* spores while other studies investigated rather the activity of selected essential oils components or plant extracts against *A. acidoterrestris* vegetative cells and spores. Maldonado et al. [31] reported that lemon oil completely inhibited alicyclobacilli spores germination within 11 days, both in laboratory medium and lemon juice concentrate. On the contrary, other studies claim that limonene (component of citrus essential oils) did not inhibit *A. acidoterrestris* spores’ outgrowth [30,38]. It was reported that the addition of eugenol and cinnamalaldehyde (20:40-ppm) into apple juice prevented spores germination for 7 day storage [29]. Similarly, the susceptibility of *A. acidoterrestris* to the eucalyptus leaves methanol-dichloromethane extracts, grape seed extracts (proanthocyanidins), lemon extract (Spencer Food Industrial, The Netherlands), neroli extract (Sigma-Aldrich, Italy), citrus extract (Biocitro^®^, Quinabra, Spain), pomegranate juice (ASYA Fruit Juice and Food Ind. Inc. Turkey), commercial rosemary extract (V20, V40), pepper family plants (*Piper peltatum* and *Piper marginatum*) hexane-dichloromethane extracts was confirmed [28,39,40,41,42,43].

Concerning the biofilm itself, Anjos et al. [13] evaluated formation of *A. acidoterrestris* biofilm on the surface of stainless steel, polyvinyl chloride, and nylon. It was found that the greatest amount of biofilm was formed on the nylon surface, which is a hydrophilic material, whereas the lowest one on the hydrophobic PVC surface. In our research, the biofilm was associated with its height and the surface structure (surface area difference) and both tested *A. acidoterrestris* strains formed greater biofilms on the hydrophobic surface (PVC) rather than the hydrophilic one (glass), what is not in agreement with Anjos et al. [13]. This suggests that a material’s surface hydrophobicity may not be a crucial factor determining the adhesion of *A. acidoterrestris* to abiotic surfaces. Both reference and environmental strain formed biofilm on the surface of PVC and glass after 72 h incubation in static as well as in agitated cultures. These results are in conformity with Shemesh et al. [14] who reported that acidothermophilic bacteria *A. acidoterrestris* generate biofilms on polystyrene and glass surfaces after 3-day incubation. Furthermore, according to Basson et al. [44] cells’ adhesion and biofilm formation is not always enhanced by the medium flow. In our research both the agitation and the tested material seemed to affect *A. acidoterrestris* ability to form biofilm. The average biofilm height and surface structure in agitated cultures were 33.5% (*A. acidoterrestris* DSM 3922) and 38.9% (*A. acidoterrestris* 055) lower for the polyvinyl chloride, whereas lack of agitation facilitated intensive glass colonization in the cultures with the clove oil.

Bacterial biofilms are non-homogenous structures characterized by a high surface roughness, diversification of cells metabolic activity, extracellular polymeric substance presence as well as cells’ and spores’ liberation from the upper parts of biofilm matrix. In our research, *A. acidoterrestris* biofilm formed in a medium supplemented with clove essential oil, composed of either a typical but loose structure or aggregates of spores and cells in an EPS layer. Additionally, atomic force microscopy imaging confirmed the presence of alicyclobacilli spores within the grown biofilms. Spore production is generally recognized as a response to unfavorable environmental conditions. Therefore, the occurrence of spores in cultures subjected to stress factors like essential oil addition or nutrients deficiency is not surprising [45]. According to Lindsay et al. [45], a surface’s colonization and biofilm formation by sporeformers occur prior to spore production. This fact is of a high importance for the food industry, as the susceptibility of bacterial biofilms to bioactive substances (bacteriocins, essential oils, plant extracts) could be greater before the massive spore production phase. Furthermore, several researches admit that *A. acidoterrestris* cells and spores proneness to active compounds differs substantially [46,47,48,49,50]. In the research proceeded by Anjos et al. [13] it was found that peracetic acid (500–1000 ppm), sodium hypochlorite (1000–2000 ppm), and quaternary ammonia-benzalkonium chloride (15.62–31.24 ppm) solutions reduced *A. acidoterrestris* biofilms on stainless steel, PVC and nylon surfaces by 1.90–5.72 log CFU/cm^2^. Comparable in our study, clove oil (0.05% which is equal to 500 ppm) reduced the level of formed biofilm on glass and PVC by 25.1–65.0% (1.07–2.74 log CFU/cm^2^), dependently on the culture condition, the tested material surface, and the strain used. The clove oil is recognized as one of the most bioactive essential oils due to high antimicrobial activity of eugenol [51]. The main compound of the clove oil used in the presented research was eugenol constituting 86.99% of oil components. The action of the clove oil against *Alicyclobacillus* biofilm may be attributed both to its bactericidal effect resulting in a decrease in the number of planktonic cells and the changes in the cells adherence capability. As it was shown in our research, the clove oil may also act as the agent causing release the EPS from biofilm and inducing detachment of bacteria from the surface. The vivid increase in the ratio of non-adherent cells to sessile cells in the biofilms formed at the presence of clove oil indicates the reduced ability of *A. acidoterrestris* cells to colonize the tested technical surfaces enduringly.

## 4. Materials and Methods

### 4.1. Microbial Strains

A reference strain *A. acidoterrestris* DSM 3922 (Deutche Sammlung von Microorganismen und Zellkulturen DSMZ, Braunschweig, Germany) and an environmental strain *A. acidoterrestis* 055, isolated from Polish apple orchard soil located near the city of Łódź, were used in the study. *A. acidoterrestis* 055 isolate was previously identified by the molecular method on the basis of 16S rRNA gene sequencing and the genomic sequence of was deposited in the GenBank database under KY045828 accession number. Bacterial strains were activated as reported by Tyfa et al. [15]. Vegetative cells of 24-h cultures grown on *Bacillus acidoterrestris* agar (BAT agar, pH 4.0; Merck, Darmstadt, Germany) were collected by swabbing and standardized in BAT broth (pH 4.0; Merck, Darmstadt, Germany), to final inoculum of approximately 4.0 log CFU units per milliliter (DEN-1 densitometer; Biosan Ltd., Riga, Latvia). The absence of spores was confirmed by staining with malachite green.

### 4.2. Clove Essential Oil and Its Chemical Analysis

The clove buds (*Syzygium aromaticum* (L.) Merr. and Perry) commercial essential oil was purchased from Pollena Aroma S.A. (Warszawa, Poland). The oil’s composition was analyzed by gas chromatography-mass spectroscopy (GC-MS) using Trace GC Ultra chromatograph (Thermo Electron Corporation, Waltham, MA, USA) equipped with Rtx-1 (Restek, Bellefonte, PA, USA) non-polar capillary column (60 m × 0,25 mm; 0,25 μm film thickness) combined with DSQ II mass spectrometer (Thermo Electron Corporation, Waltham, MA, USA), according to the procedure described by Smigielski et al. [52]. The temperature was programmed as follows: 50–300 °C at 4 °C/min; injector (SSL) temperature 280 °C; the detector (FID) temperature 300 °C; carrier gas helium with constant pressure 300 kPa; split ratio 1:37. The mass spectrometer operating parameters: Ion source temperature 200 °C; ionization energy 70 eV (EI). The identification of the oil’s components was based on the comparison of their retention indices (RI), mass spectra (NIST and Wiley libraries) and the literature [53].

### 4.3. Preparation of Abiotic Surfaces, Biofilm Growth Conditions, and Quantification

The glass slides (Bionovo, Legnica, Poland) and polyvinyl chloride (PVC) slides (Simona, Kirn, Germany) (25 × 80 mm^2^) were used as the surface tested. Each selected material was individually washed and sterilized according to the technique described by Marques et al. [54] with modifications. Both materials were sanitized with Bacticid disinfectant (Chemi Pharm AS, Tallinn, Estonia), rinsed with sterile distilled water twice and air-dried. Afterwards, the slides were sanitized with ethanol solution (96% *v/v*), rinsed with sterile distilled water and dried for 2 h in 60 °C. Finally, the material was sterilized for 15 min in the temperature 121 °C (glass) or in ultraviolet light (λ 254 nm) for 30 min (PVC). The glass or PVC slides were placed in the sterile glass bottle containing 49.5 mL BAT broth (pH 4.0) and 500 µL of vegetative *A. acidoterrestris* cells suspension in BAT broth. The cultures were incubated in 44 °C for 72 h both with (60 rpm) and without agitation (static). Simultaneously, the cultures with an addition of 0.05% (*v/v*) clove oil were prepared and incubated analogously. The biofilm formed on the technical materials was collected by swabbing. Simultaneously, loosely attached cells (non-adherent cells) were rinsed with saline solution (0.85% NaCl) and the remaining adhered cells (sessile cells) were collected by swabbing. The levels of biofilm, non-adherent cells and sessile cells were estimated by the plate count method on BAT agar medium (incubation 44 °C, 72 h). The experiments were conducted in triplicate and results presented as log CFU/cm^2^.

### 4.4. Biofilm Surface Characterization by AFM

The morphology of biofilms formed on glass and PVC surfaces was examined by atomic force microscopy (AFM). AFM measurements were performed in air using a Bioscope II AFM with a NanoScope V controller (Veeco, Santa Barbara, CA, USA). Biofilms were imaged in contact mode using an MLCT-D silicon nitride cantilever with a nominal tip apex radius of 20 nm. The height and deflection images were obtained simultaneously at a scan rate 0.5 Hz, at a resolution of 512 pixels per line. Each image has been done in different places selected randomly from 30 × 30 µm^2^ area, for a sample. AFM images were flattened and plane fitted prior to analysis. The calculated surface characteristics parameters included the root mean square (RMS) roughness (Rq), average height and surface area differences. The Rq is the root mean square average of height deviations taken from the mean data plane. Average height is the average of all the Z values. Surface area difference is the difference between the analyzed region’s three dimensional surface area and its two dimensional projected surface area. The data were analyzed with the NanoScope Analysis 1.7 software from Bruker. AFM imaging was performed in the Center of Quantum Optics at the Faculty of Physics, Astronomy and Informatics, Nicolaus Copernicus University in Toruń, Poland.

### 4.5. Statistical Analysis

The final results of AFM and biofilm data were shown as the Root Mean Square along with standard deviation (±SD).

## 5. Conclusions

The medium-flow and the type of abiotic surface have a significant influence on *A. acidoterrestris* biofilm development. The clove oil inhibited the biofilm formation and led to changes in its structure. The oil in concentration of 0.05% caused a substantial reduction of the biofilm formed both on glass and polyvinyl chloride surfaces. Additionally, clove oil induced alteration in bacterial morphology, associated with changes in the cells length or visible increase of its roughness. Moreover, clove essential oil may not only release the EPS from biofilm inducing detachment of bacteria from surface, but also affect the adherence capability of cells. Notwithstanding, the use of clove oil to minimize the *A. acidoterrestris* biofilm development in industrial practice may carry a risk of impacting essential oil residue constituents on taste of food products. Since *A. acidoterrestris* is mainly a threat to the production of fruit juices, their contact with traces of clove oil, with an organoleptic profile consistent with a number of juices such as apple or citrus, should not significantly affect the taste of the product, and may even slightly enrich its aroma. The clove oil may serve a potential antibiofilm agent in fruit juice industry. The potential use of clove essential oil, e.g., as an additive to sluicing water, may hinder a development of *A. acidoterrestris* biofilms on production surfaces, which will be further investigated.

## Figures and Tables

**Table 1:** Chemical composition and contents of principal component of clove oil.

Compound	RI	Concentration (%)
Eugenol	1346	86.99 ± 0.09
*α*- Copaene	1377	00.07 ± 0.01
*β*-Caryophyllene	1422	08.76 ± 0.04
Cadina-1(6),4-diene	1468	00.04 ± 0.01
*α*-Humulene	1452	01.91 ± 0.03
*ɣ*-Muurolene	1470	00.02 ± 0.01
*β*-Selinene	1482	00.02 ± 0.01
*α*-Selinene	1491	00.05 ± 0.02
*β*-Farnesene	1494	00.06 ± 0.01
(Z)-Calamenene	1509	00.11 ± 0.02
δ-Cadinene	1513	00.27 ± 0.03
Cadina-1,4-diene	1524	00.03 ± 0.01
Humulene epoxide	1594	00.11 ± 0.01
Total		98.44

## References

[1] Abee T., Kovács Á.T., Kuipers O.P., van der Veen S. (2011). Biofilm formation and dispersal in Gram-positive bacteria. Curr. Opin. Biotech..

[2] Bogino P.C., Oliva M.D.M., Sorroche F.G., Giordano W. (2013). The role of bacterial biofilms and surface components in plant-bacterial associations. Int. J. Mol. Sci..

[3] Czaczyk K., Myszka K. (2007). Biosynthesis of extracellular polymeric substances (EPS) and its role in microbial biofilm formation. Pol. J. Environ. Stud..

[4] Vu B., Chen M., Crawford R.J., Ivanova E.P. (2009). Bacterial extracellular polysaccharides involved in biofilm formation. Molecules.

[5] Auger S., Ramarao N., Faille C., Fouet A., Aymerich S., Gohar M. (2009). Biofilm formation and cell surface properties among pathogenic and nonpathogenic strains of the *Bacillus cereus* group. Appl. Environ. Microbiol..

[6] Galié S., García-Gutiérrez C., Miguélez E.M., Villar C.J., Lombó F. (2018). Biofilms in the food industry: Health aspects and control methods. Front. Microbiol..

[7] Han Q., Song X., Zhang Z., Fu J., Wang X., Malakar P.K., Liu H., Pan Y., Zhao Y. (2017). Removal of foodborne pathogen biofilms by acidic electrolyzed water. Front. Microbiol..

[8] Srey S., Jahid I.K., Ha S.D. (2013). Biofilm formation in food industries: A food safety concern. Food Control.

[9] Steyn C.E., Cameron M., Witthuhn R.C. (2011). Occurrence of *Alicyclobacillus* in the fruit processing environment—A review. Int. J. Food Microbiol..

[10] Danyluk M.D., Friedrich L.M., Jouquand C., Goodrich-Schneider R., Parish M.E., Rouseff R. (2011). Prevalence, concentration, spoilage, and mitigation of *Alicyclobacillus* spp. in tropical and subtropical fruit juice concentrates. Food Microbiol..

[11] McKnight I.C., Eiroa M.N.U., Sant’Ana A.S., Massaguer P.R. (2010). *Alicyclobacillus acidoterrestris* in pasteurized exotic Brazilian fruit juices: Isolation, genotypic characterization and heat resistance. Food Microbiol..

[12] Zhang J., Yue T., Yuan Y. (2013). *Alicyclobacillus* contamination in the production line of kiwi products in China. PLoS ONE.

[13] Anjos M.M., dos Ruiz S.P., Nakamura C.V., de Abreu Filho B.A. (2013). Resistance of *Alicyclobacillus acidoterrestris* spores and biofilm to industrial sanitizers. J. Food Protect..

[14] Shemesh M., Pasvolsky R., Zakin V. (2014). External pH is a cue for the behavioral switch that determines surface motility and biofilm formation of *Alicyclobacillus acidoterrestris*. J. Food Prot..

[15] Tyfa A., Kunicka-Styczyńska A., Zabielska J. (2015). Evaluation of hydrophobicity and quantitative analysis of biofilm formation by *Alicyclobacillus* sp.. Acta Biochim. Pol..

[16] Fu Y., Zu Y., Chen L., Shi X., Wang Z., Sun Z., Effert T. (2007). Antimicrobial activity of clove and rosemary essential oils alone and in combination. Phytother. Res..

[17] Nuñez L., D’Aquino M. (2012). Microbicide activity of clove essential oil (*Eugenia caryophyllata*). Braz. J. Microbiol..

[18] Xu J.G., Liu T., Hu Q.P., Cao X.M. (2016). Chemical composition, antibacterial properties and mechanism of action of essential oil from clove buds against *Staphylococcus aureus*. Molecules.

[19] Gupta C., Garg A., Uniyal R., Gupta S. (2009). Comparison of antimicrobial activities of clove oil and its extract on some food borne microbes. Internet J. Microbiol..

[20] Pinto E., Vale-Silva L., Cavaleiro C., Salgueiro L. (2009). Antifungal activity of the clove essential oil from *Syzygium aromaticum* on *Candida*, *Aspergillus* and dermatophyte species. J. Med. Microbiol..

[21] Hoque M.M., Bari M.L., Juneja V.K., Kawamoto S. (2008). Antimicrobial activity of cloves and cinnamon extracts against food borne pathogens and spoilage bacteria, and inactivation of *Listeria monocytogenes* in ground chicken meat with their essential oils. Rep. National Food Res. Inst..

[22] Kim Y.-G., Lee J.-H., Gwon G., Kim S.-I., Park J.G., Lee J. (2016). Essential oils and eugenols inhibit biofilm formation and the virulence of *Escherichia coli* O157:H7. Sci. Rep..

[23] Husain F.M., Ahmad I., Asif M., Tahseen Q. (2013). Influence of clove oil on certain quorum-sensing-regulated functions and biofilm of *Pseudomonas aeruginosa* and *Aeromonas hydrophila*. J. Biosci..

[24] Cui H., Zhang C., Li C., Lin L. (2020). Inhibition of *Escherichia coli* O157:H7 biofilm on vegetable surface by solid liposomes of clove oil. LWT.

[25] Sellamuthu R. (2014). Eugenol. Encyclopedia of Toxicology. Reference Module in Biomedical Sciences.

[26] Nowak K., Ogonowski J., Jaworska M., Grzesik K. (2012). Clove oil—Properties and applications. Chemik.

[27] Bevilacqua A., Corbo M.R., Sinigaglia M. (2011). Use of essential oils to inhibit *Alicyclobacillus acidoterrestris*: A short overview of the literature. Front. Microbiol..

[28] Bevilacqua A., Campaniello D., Speranza B., Sinigaglia M., Corbo M.R. (2013). Control of *Alicyclobacillus acidoterrestris* in apple juice by citrus extracts and a mild heat-treatment. Food Control.

[29] Bevilacqua A., Corbo M.R., Sinigaglia M. (2010). Combining eugenol and cinnamaldehyde to control the growth of *Alicyclobacillus acidoterrestris*. Food Control.

[30] Huertas J.P., Esteban M.D., Antolinos V., Palop A. (2014). Combined effect of natural antimicrobials and thermal treatments on *Alicyclobacillus acidoterrestris* spores. Food Control.

[31] Maldonado M.C., Aban M.P., Navarro A.R. (2013). Chemicals and lemon essential oil effect on *Alicyclobacillus acidoterrestris* viability. Braz. J. Microbiol..

[32] Orr R.V., Beuchat L.R. (2000). Efficacy of disinfectants in killing spores of *Alicyclobacillus acidoterrestris* and performance of media for supporting colony development by survivors. J. Food Prot..

[33] Podolak R., Elliott P.H., Taylor B.J., Khurana A., Black D.G. (2009). Destruction of *Alicyclobacillus acidoterrestris* spores in apple juice on stainless steel surfaces by chemical disinfectants. J. Food Prot..

[34] Tyfa A., Kunicka-Styczyńska A., Dąbrowska J. (2015). Activity of compounds of natural origin against *Alicyclobacillus acidoterrestris*, a common fruit juices contaminant. Biotechnol. Food Sci..

[35] (2005). European Pharmacopoeia.

[36] Prado D.B., dos Anjos Szczerepa M.M., Capeloto O.A., Astrath N.G.C., dos Santos N.C.A., Previdelli I.T.S., Nakamura C.V., Mikcha J.M.G., Abreu Filho B.A. (2019). Effect of ultraviolet (UV-C) radiation on spores and biofilms of *Alicyclobacillus* spp. in industrialized orange juice. Int. J. Food Microbiol..

[37] Prado D.B., Fernandes M.S., Anjos M.M., Tognim M.C.B., Nakamura C.V., Machinski Jr M., Mikcha J.M.G., Abreu Filho B.A. (2018). Biofilm-forming ability of *Alicyclobacillus* spp. isolates from orange juice concentrate processing plant. J. Food Safety.

[38] Bevilacqua A., Corbo M.R., Sinigaglia M. (2008). Inhibition of *Alicyclobacillus acidoterrestris* spores by natural compounds. Int. J. Food Sci. Technol..

[39] Molva C., Baysal A. (2015). Antimicrobial activity of grape seed extract on *Alicyclobacillus acidoterrestris* DSM 3922 vegetative cells and spores in apple juice. Food Sci. Technol..

[40] Molva C., Baysal A.H. (2015). Evaluation of bioactivity of pomegranate fruit extract against *Alicyclobacillus acidoterrestris* DSM 3922 vegetative cells and spores in apple juice. Food Sci. Technol..

[41] de Pascoli I.C., dos Anjos M.M., da Silva A.A., Lorenzetti F.B., Cortez D.A.G., Mikcha J.M.G., Nakamura T.U., Nakamura C.V., de Abreu Filho B.A. (2018). Piperaceae extracts for controlling *Alicyclobacillus acidoterrestris* growth in commercial orange juice. Ind. Crop. Prod..

[42] Piskernik S., Klančnik A., Demšar L., Smole Možina S., Jeršek B. (2016). Control of *Alicyclobacillus* spp. vegetative cells and spores in apple juice with rosemary extracts. Food Control.

[43] Takahashi T., Kokubo R., Sakaino M. (2004). Antimicrobial activities of eucalyptus leaf extracts and flavonoids from *Eucalyptus maculata*. Lett. Appl. Microbiol..

[44] Basson A., Flemming L.A., Chenia H.Y. (2008). Evaluation of adherence, hydrophobicity, aggregation, and biofilm development of *Flavobacterium johnsoniae*-like isolates. Microb. Ecol..

[45] Lindsay D., Brözel V.S., von Holy A. (2005). Spore formation in *Bacillus subtilis* biofilms. J. Food Protect..

[46] Bevilacqua A., Ciuffreda E., Sinigaglia M., Corbo M.R. (2014). Effects of lysozyme on *Alicyclobacillus acidoterrestris* under laboratory conditions. Food Sci. Technol..

[47] De Carvalho A.A.T., Vanetti M.C.D., Mantovani H.C. (2008). Bovicin HC5 reduces thermal resistance of *Alicyclobacillus acidoterrestris* in acidic mango pulp. J. Appl. Microbiol..

[48] Molva C., Baysal A.H. (2017). Modeling growth of *Alicyclobacillus acidoterrestris* DSM 3922 type strain vegetative cells in the apple juice with nisin and lysozyme. Microbiology.

[49] Pei J., Yue T., Yuan Y. (2014). Control of *Alicyclobacillus acidoterrestris* in fruit juices by a newly discovered bacteriocin. World J. Microb. Biot..

[50] Yamazaki K., Murakami M., Inoue N., Matsuda T. (2000). Use of nisin for inhibition of *Alicyclobacillus acidoterrestris* in acidic drinks. Food Microbiol..

[51] Kalemba D., Kunicka A. (2003). Antibacterial and antifungal properties of essential oils. Curr. Med. Chem..

[52] Smigielski K., Raj A., Krosowiak K., Gruska R. (2009). Chemical composition of the essential oil of *Lavandula angustifolia* cultivated in Poland. J. Essent. Oil Bear. Plants.

[53] Adams R.P. (2007). Identification of Essential Oil Components by Gas Chromatography/Mass Spectroscopy.

[54] Marques S.C., Rezende J.D.G.O.S., Alves L.A.D.F., Silva B.C., Alves E., Abreu L.R.D., Piccoli R.H. (2007). Formation of biofilms by *Staphylococcus aureus* on stainless steel and glass surfaces and its resistance to some selected chemical sanitizers. Braz. J. Microbiol..

